# In sync with the heart

**DOI:** 10.7554/eLife.84298

**Published:** 2022-11-17

**Authors:** Aleksandra M Herman

**Affiliations:** 1 Laboratory of Brain Imaging, Nencki Institute of Experimental Biology, Polish Academy of Sciences Warsaw Poland

**Keywords:** active sensing, tactile discrimination, interoception, cardiac cycle, active inference, touch, Human

## Abstract

People actively adjust how they acquire sensory information, such as tactile cues, based on how their bodily functions alter their senses.

**Related research article** Galvez-Pol A, Virdee P, Villacampa J, Kilner J. 2022. Active tactile discrimination is coupled with and modulated by the cardiac cycle. *eLife* **11**:e78126. doi: 10.7554/eLife.78126.

Sit back and relax. Close your eyes. Can you feel your heart beating in your chest? What you are experiencing is your heart working in a cyclic manner. During the systolic phase, the heart contracts, ejecting blood into the vessels that lead out of it, and increasing the activity of pressure sensors called baroreceptors. During the diastolic phase, the heart expands, allowing blood to flow into it, while the baroreceptors remain quiescent.

Even though the whole cycle usually takes just under a second, a lot is happening during that time. With every heartbeat, the brain receives input about the strength and precise timing of each cardiac contraction – information that needs to be promptly processed and acted upon if necessary. In recent years, a lot of studies have focused on the internal state of our body and the way we process its subtle signals; and in how physiological fluctuations in our bodies (such as the cardiac cycle) can impact our cognition and behaviour.

Certain behaviours have been shown to occur in sync with internal bodily oscillations (Kunzendorf et al., 2019; Ohl et al., 2016). For example, when we look for something, we fixate our eyes more (i.e., sample new information) during diastole, and move our eyes more (to search new areas) during systole (Galvez-Pol et al., 2020). Moreover, the sensitivity of touch also varies between the two phases: people are less perceptive to touch during systole than during diastole (Al et al., 2020; Motyka et al., 2019). So far, it was unclear what behavioural benefits such synchronicity brings (Herman and Tsakiris, 2021). Now, in eLife, Alejandro Galvez-Pol, Pavandeep Virdee, Javier Villacampa and James Kilner of University College London and the University of the Balearic Islands report new insights on this matter (Galvez-Pol et al., 2022).

In a cleverly designed experiment, Galvez-Pol et al. used electrocardiography to record the cardiac activity of participants while they performed a simple tactile discrimination task. Without looking, the participants had to figure out whether the objects they touched had vertical or horizontal grooves. They found that touches initiated during systole were held for longer than touches initiated during diastole (Figure 1). This was particularly pronounced when it was difficult to discriminate the objects, indicating that people use prolonged touch to compensate for the reduced sensitivity during systole.

**Figure 1.**
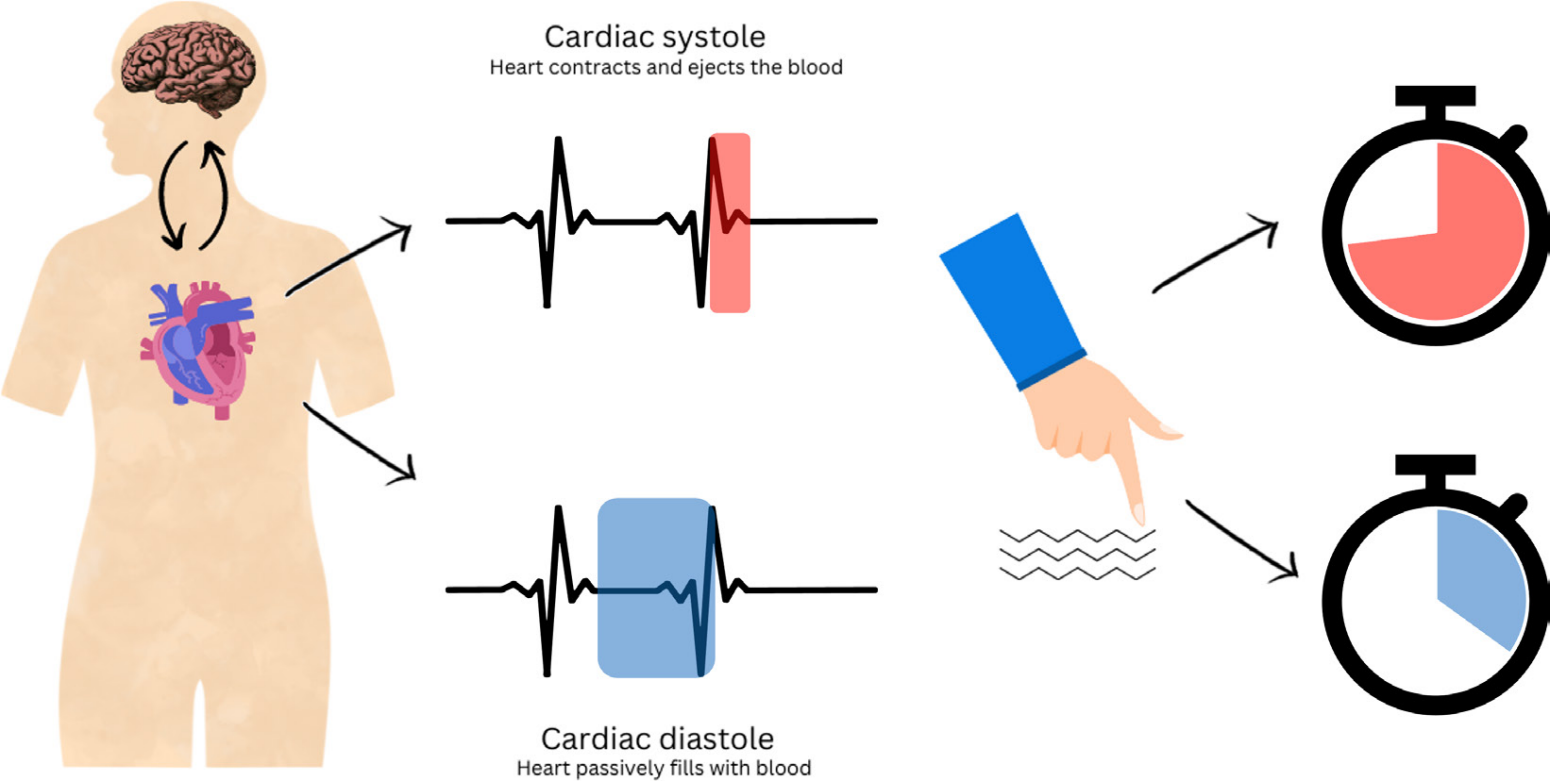
Schematic representation of the interplay between cardiac cycles and perception. The heart beats in a cyclic manner. It contracts to actively push blood around the body (systolic phase, red, top centre) and relaxes to refill again (diastolic phase, blue, bottom centre). Using a tactile discrimination task, Galvez-Pol et al. show that the sensitivity of touch decreases during systole: therefore, to compensate, people hold their fingers longer over an object (red clock), especially when the task was more difficult. Conversely, people will hold their fingers on an object for a shorter time if they start touching it during diastole (blue clock).

Moreover, Galvez-Pol et al. found that the timing of touch also affected the duration of a cardiac cycle. When touch was initiated during systole, it increased the proportion of the cycle in diastole, which had previously been associated with the greatest tactile sensitivity. Thus, people adapt their behaviour in line with their perceptual needs; but their internal bodily cycles also adjust according to external demands to ensure a stable perception of the world around us.

Our bodies work in a rhythmic fashion – a fact that we typically pay little attention to in our daily lives. However, these internal rhythms have a much greater influence on our cognition than previously thought and can modulate how we perceive the environment around us. But there is still a lot to discover. Bodily signals may impact our perception in a relatively simple tactile discrimination task, but do they also affect more complex cognitive processes, such as decision-making? And are individuals, who are more attuned to subtle changes in their physiology, better able to adjust their behaviours to overcome cardiac-related effects? Answering these questions will allow us to better understand the complex interplay between the brain and the rest of the body and, ultimately, better understand ourselves.
